# Anterior cruciate ligament reconstruction: a new cortical suspension device for femoral fixation with transtibial and transportal techniques

**DOI:** 10.1186/s13018-014-0110-7

**Published:** 2014-11-19

**Authors:** Luiz Gabriel Betoni Guglielmetti, Ricardo de Paula Leite Cury, Victor Marques de Oliveira, Osmar Pedro Arbix de Camargo, Nilson Roberto Severino, Patrícia Maria de Moraes Barros Fucs

**Affiliations:** Department of Orthopedics and Traumatology, Santa Casa Medical School and Hospitals, R. Dr. Cesário Motta Junior, 112, São Paulo, 01221-020 Brazil

**Keywords:** Anterior cruciate ligament, Orthopedic fixation devices, Arthroscopy, Knee

## Abstract

**Background:**

In the field of anterior cruciate ligament (ACL) reconstruction, there is still no consensus regarding the proper fixation method and position of the tunnels. The primary objective of this paper was to describe a new fixation device, the Endo Tunnel Device (ETD®), for both techniques (transtibial and transportal), as well as the associated difficulties and the intraoperative and postoperative intercurrences. The secondary objective was to describe a preliminary clinical evaluation (6 months of follow-up) comparing these techniques.

**Methods:**

This was a prospective, randomized study involving 80 patients with ACL reconstructions using the ETD® for femoral fixation. Forty patients underwent the transtibial technique, and 40 patients underwent the transportal technique. Patients were evaluated by radiography, physical examination, the KT1000 arthrometer, and Lysholm and the International Knee Documentation Committee (IKDC) scores.

**Results:**

There were more intraoperative intercurrences in the transportal group (soft tissue device fixation, short femoral tunnel, and short graft inside the tunnel). The IKDC scores were significantly better in the transportal group.

**Conclusions:**

The ETD® was demonstrated to be a safe femoral fixation device in this trial; its use in both the transtibial and transportal techniques is technically simple and is associated with few intra- or postoperative complications.

## Introduction

Anterior cruciate ligament (ACL) reconstruction is one of the most commonly performed surgeries in orthopedics [1]. Many femoral fixation methods can be used for hamstring tendon grafts. Several studies have compared fixation methods, and although the methods show mechanical differences in laboratory studies [2-4], they are clinically similar when correctly used [5-7].

The femoral tunnel can be drilled using a guide through the tibial tunnel, by using the outside-in technique, or via the medial portal. In the last two decades, the most commonly used method worldwide has been the transtibial technique [8]. However, anatomical studies have shown that with this technique, the tunnel is not positioned in the center of the origin of the ACL [9,10] and other biomechanical [10-12] and clinical studies [13,14] have shown advantages with regard to the stability gained with the more anatomical position of the femoral tunnel.

The Endo Tunnel Device (ETD®; ProInd, Cotia, São Paulo, Brazil) is a metallic implant used for femoral fixation of the semitendinosus and gracilis autografts for ACL reconstruction. This implant was developed in 2006 and has been used in Brazil since 2007. The device can be used with both the transtibial technique and the anatomical technique. No studies on this device have been published.

The primary objective of this study was to assess the technical aspects, difficulties, and intraoperative and postoperative intercurrences of the use of the ETD® with the transtibial and transportal techniques. The secondary objective was to perform a preliminary clinical evaluation (6 months of follow-up) of these techniques (transtibial X transportal). The final clinical evaluation, after a 2-year follow-up, is the objective of another study that is ongoing.

## Methods

Between August 2010 and May 2012, 145 patients underwent ACL reconstruction in the Knee Clinic at the Orthopedics Department of Santa Casa Medical School and Hospitals in São Paulo, Brazil. Among these patients, 80 fulfilled the inclusion criteria of this study, namely, unilateral ACL lesion, skeletally mature with closed physis, age less than 40 years, no previous surgery on the affected knee (except for arthroscopic meniscectomy), no degenerative changes on arthroscopy, less than 1 year since injury, no associated ligament injuries (except for medial collateral ligament tears of grades I and II), and no morbid obesity.

All patients were operated on by the same surgeon or under his supervision. They were recruited prospectively according to a protocol that was previously approved by the hospital’s ethics committee (Comitê de Ética em Pesquisa da Irmandade da Santa Casa de Misericórdia de São Paulo). Once included in the study, the patients were randomized to one of two groups: the “transtibial group,” consisting of patients undergoing femoral tunnel drilling with the transtibial technique, and the “transportal group,” consisting of patients undergoing the transportal technique. The randomization was performed by selecting a folded piece of paper that was placed in opaque envelopes with the group number (1-transtibial, 2-transportal). There were 40 pieces of paper with the number 1 and 40 pieces of paper with the number 2; after removal, each piece of paper was not replaced. This randomization was designed for another study with a different primary objective, namely, a clinical evaluation comparing these two groups with 2 years of follow-up. The other study is currently ongoing. None of the assessors were blinded.

The patients were preoperatively evaluated by a KT1000 arthrometer (MEDmetric, San Diego, California) at 20° of flexion in response to a 133-N load, Lachman test, anterior drawer test, pivot-shift test, objective International Knee Documentation Committee (IKDC) score [15], subjective IKDC score, and Lysholm test [16]. The final clinical evaluation after 2 years has not yet been completed.

For both groups, the gracilis and semitendinosus tendons were used as grafts. No case was treated with selective reconstruction of only one of the ACL bundles. The tibial tunnel was created with the knee in extension using a Howell™ 65° Tibial Guide (Biomet Sports Medicine Inc., Warsaw, Indiana). A metal interference screw was placed on the tibial fixation.

For the femoral tunnel in the transtibial group, we used a transtibial aimer placed in the posterior intercondylar margin. In the transportal group, after removal of the ACL remains and identification of the origin and insertion of the ligament, the chondral picker or “icepicker” was inserted, and the center of the native ACL was marked. A guide wire was inserted and positioned on the previously marked point, the knee was flexed by 120° to 130°, and finally, the guide wire was advanced until it passed through the lateral cortex of the femur. The femoral tunnel was then drilled. After measuring the length of the femoral tunnel, the appropriate size of the ETD® was selected. Femoral fixation with the ETD® was performed in both groups of patients.

**Figure 1:**
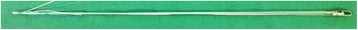
**Photograph of an Endo Tunnel Device (ETD®).**

The ETD® (Figure 1) is a femoral fixation system for reconstruction of the ACL with hamstrings. It is a titanium alloy implant (Ti6AI4V), and according to the ASTMF136 specifications, it is capable of supporting an average load of 97.7 kgf. It should be fixed to the lateral femoral cortex using a moving metal lever within the cylindrical structure of the implant. Once the implant is outside the lateral cortex, the wire is pulled into the two holes in the ends of the lever, positioning the lever perpendicular to the cylindrical structure. By pulling the graft distally, this lever fixes the device in the lateral femoral cortex. The graft is fixed on the distal end of the cylindrical part, which is composed of a metal strap fixed to the cylindrical body.

**Figure 2:**
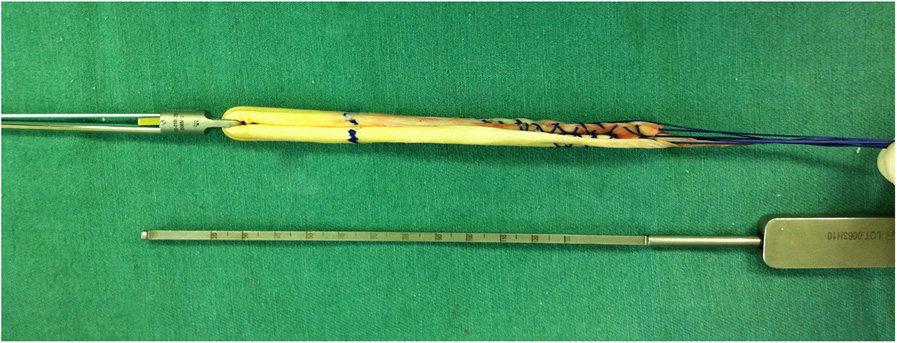
**Photograph of the prepared graft, measured and marked with methylene blue and ready for insertion.**

The implant is available in various diameters and lengths; specifically, the diameter varies from 7 to 9 mm, and the length can be 20, 25, 30, or 35 mm. The diameter of the graft determines the diameter to be used and, therefore, the tibial and femoral tunnel diameters. The femoral tunnel should be drilled such that it passes through the lateral cortex. After drilling, the length of the femoral tunnel is measured, which allows selection of the length of the ETD®. The graft is marked so the surgeon knows when to activate the fixation plate (Figure 2).

**Figure 3:**
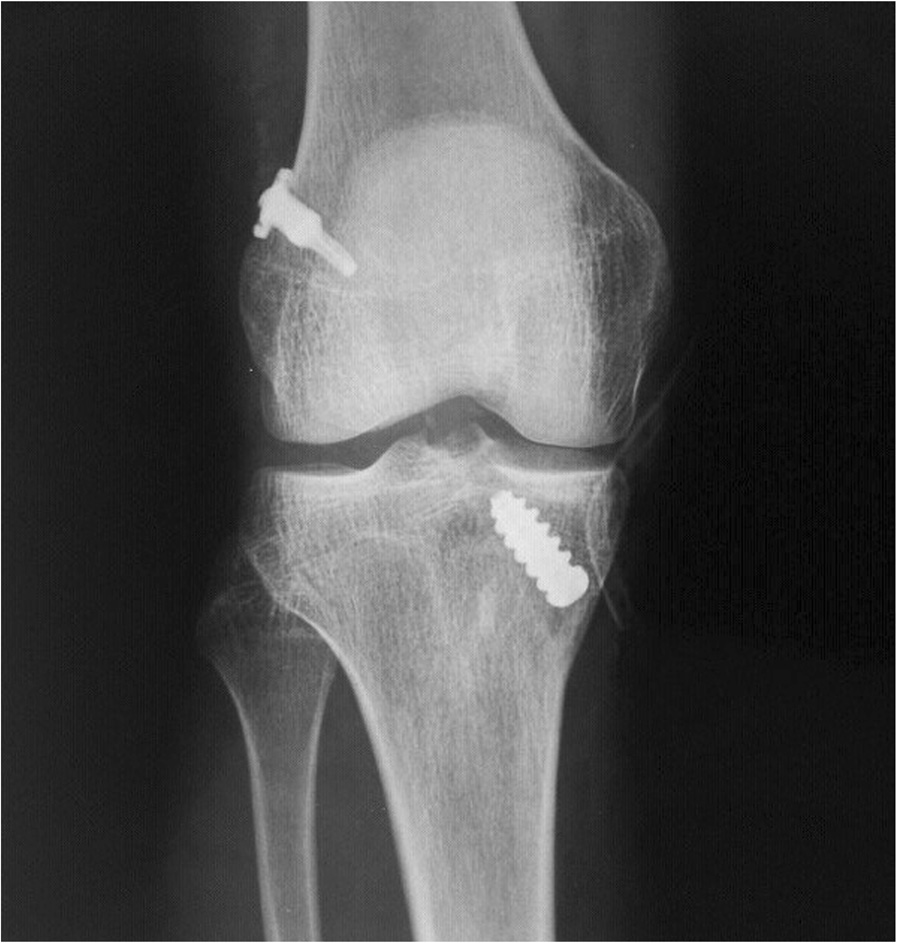
**Postoperative anterior-posterior radiograph of an anterior cruciate ligament reconstruction, showing femoral fixation with the ETD®.**

**Figure 4:**
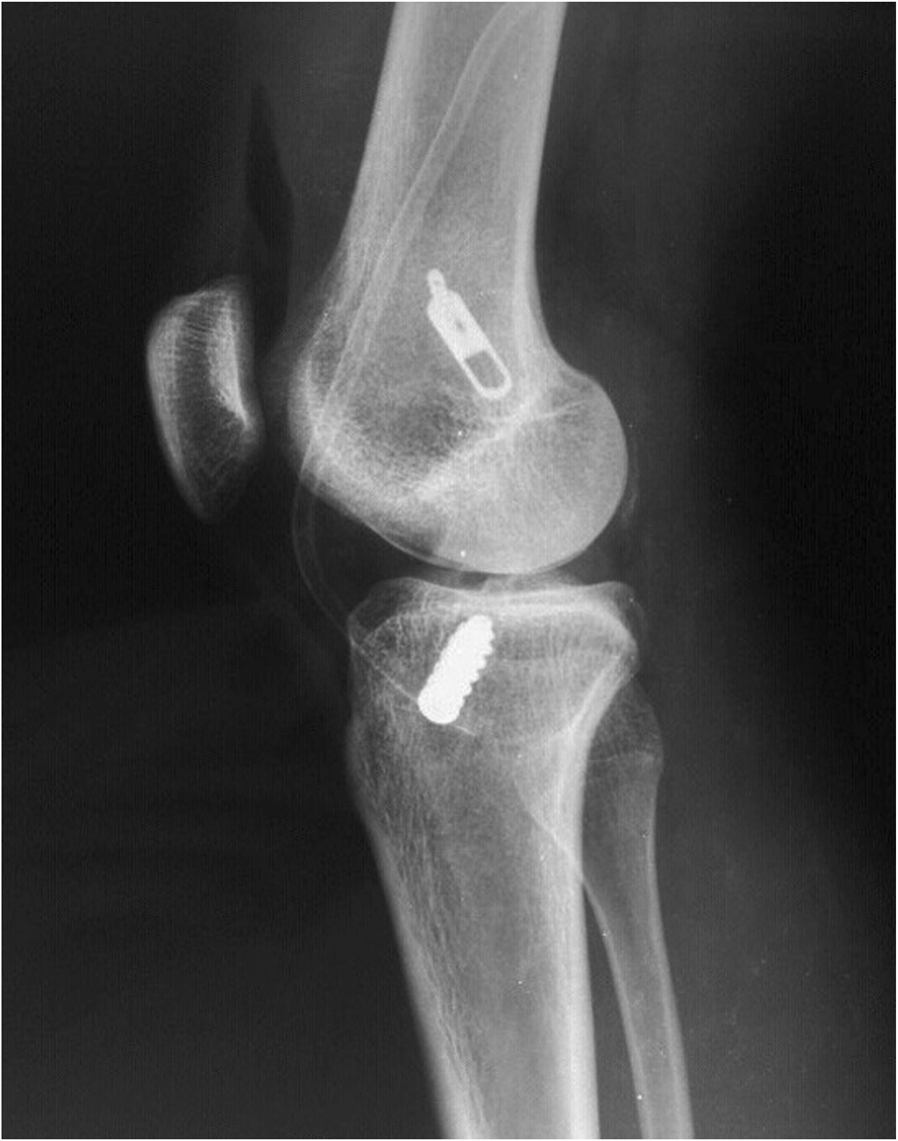
**Postoperative profile radiograph of an anterior cruciate ligament reconstruction (same case as that shown in Figure** 3 **), showing femoral fixation with the ETD®.**

A rigid metal wire attached to the cylindrical body is inserted through the medial portal or tibial tunnel, followed by the femoral tunnel, by drilling through the soft tissue and skin of the lateral thigh. After pulling the graft into the femoral tunnel, the wire drive is removed from the device, and one of the sutures attached to the proximal lever is pulled, locking it in the lateral femoral cortex, as described previously. Then, the graft is fixed in the tibial tunnel with an interference screw (Figures 3 and 4).

All patients received the same physical therapy protocol.

Statistical analysis was performed with the SPSS version 13.0 software for Windows (SPSS Inc., Chicago, IL, USA), and *p* < 0.05 was chosen to indicate statistical significance.

## Results

Of the 80 patients, 59 were male and 21 were female, with surgery performed on 43 right knees and 37 left knees. The mean age of the patients was 24 years. The average time before the surgery was performed was 6.5 months; 7 patients were lost to follow-up (5 from the transtibial group and 2 from the transportal group), so 73 patients were evaluated with a follow-up of 6 months. For the intraoperative and immediate postoperative evaluations, the entire sample of 80 patients was available.

The age, gender, lesion side, preoperative subjective IKDC score, Lysholm test score, preoperative objective IKDC score, and time post-injury at which the surgery was performed were not significantly different between the groups.

The following perioperative intercurrences were considered: soft tissue fixation of the ETD®, intratunnel fixation of the ETD®, chondral lesion of the femoral condyle, rupture of the posterior cortex of the femoral tunnel, short tunnel (less than 3.5 cm), and short graft length inside the tunnel (less than 2 cm). Postoperative complications (up to 6 months) included mobility deficits, arthrofibrosis, and superficial and deep infections.

The perioperative intercurrences were evaluated in the entire sample of 80 patients. In 6 cases, the ETD® was fixed in the soft tissues, which was verified by the immediate postoperative radiograph. Among these 6 patients, 5 were from the transportal group and 1 was from the transtibial group, with no significant difference between the groups (*p* = 0.201, Fisher’s exact test). One of these patients experienced migration of the ETD®, which became fixed to the lateral cortex 2 months after surgery. In the remaining cases of fixation in the soft tissues, there was no migration. No patients who underwent ETD® fixation in the soft tissues had a positive Lachman instability test, pivot-shift, or anterior drawer findings at 6 months post-surgery.

The average length of the femoral tunnels was 4.98 cm in the transtibial group and 3.99 cm in the transportal group (*p* = 0.001, Student’s *t* test). The average graft length inside the tunnel was 2.91 cm in the transtibial group and 2.27 cm in the transportal group (*p* < 0.001, Student’s *t* test). Fixation of the ETD® inside the tunnel occurred in only one case, without migration after surgery. There was no case involving rupture of the posterior cortex of the femoral tunnel or chondral lesion in the femoral condyle.

There was one case of superficial infection in the transportal group, and the patient was treated with antibiotics only. There was no case of deep infection. Only one patient exhibited mobility deficits and arthrofibrosis, and this case was from the transportal group. Arthroscopic debridement and intensive physical therapy were sufficient for facilitating recovery of normal mobility.

Considering perioperative intercurrences, the transportal group had significantly more problems than the transtibial group (12 versus 2; *p* = 0.003, chi-square test). With regard to postoperative intercurrences, there was no significant difference between the groups (*p* = 0.616, Fisher’s exact test). There was no significant association between perioperative intercurrences and IKDC scores 6 months post-surgery (*p* = 0.353, chi-square test).

For the preliminary clinical evaluation at 6 months postoperatively, seven patients were excluded (lost to follow-up), and the remaining 73 were included in the analysis. The results of the Lachman test, pivot-shift test, anterior drawer exam, and KT1000 test at 6 months are shown in Table 1. Although there were some differences, they were not statistically significant between the transtibial and transportal groups (*p* values provided in Table 1, chi-square test).

**Table 1:** **Results of physical examinations in the transtibial (TT) and transportal (TP) groups at 6 months post-surgery**

**Technique**	**0**	**1**	**2**	**3**	**Total**	***p*** **value**
Anterior drawer						
TT	26	8	1	0	35	0.732
TP	29	9	0	0	38	
Total	55	17	1	0	73	
Lachman						
TT	25	9	1	0	35	0.103
TP	33	4	1	0	38	
Total	58	13	2	0	73	
Pivot-shift						
TT	26	8	1	0	35	0.173
TP	33	4	1	0	38	
Total	59	12	2	0	73	
Difference in KT1000 results between knees	Up to 3 mm		More than 3 mm			
TT	27		8		35	0.156
TP	34		4		38	
Total	61		12		73	

Table 2 shows the results of the objective IKDC scores. For better visualization, the results were divided into two groups: IKDC a and IKDC b, c, or d (Table 3). The objective IKDC test results were significantly different between the groups, with patients undergoing the transportal technique exhibiting better results (*p* = 0.049, chi-square test). Subjective Lysholm and IKDC scores at 2 years postoperatively will be provided in a future publication.

**Table 2:** **Objective International Knee Documentation Committee (IKDC) results (a, b, or c) at 6 months post-surgery in the transtibial (TT) and transportal (TP) groups**

**Technique**	**a**	**b**	**c**	**Total**
IKDC				
TT	18	15	2	*35*
TP	28	9	1	*38*
Total	*46*	*24*	*3*	*73*

**Table 3:** **Objective International Knee Documentation Committee (IKDC) results at 6 months post-surgery in the transtibial (TT) and transportal (TP) groups, with b and c scores combined**

**Technique**	**a**	**b and c**	**Total**
IKDC			
TT	18	17	*35*
TP	28	10	*38*
Total	*46*	*27*	*73*

## Discussion

The ETD® was first used in 2007 in our practice, and over the past 6 years, approximately 414 ACL reconstructions have been performed using the ETD® for femoral fixation. Of these reconstructions, the transtibial technique was used in most cases, the transportal technique was used in some cases, and the “outside-in” technique was used in a few cases. Currently, there are no studies in the literature on the ETD®, which was designed by Prof. Severino to produce fixation similar to that afforded by the Endobutton, but without a polyester suture connecting the graft to the cortical fixation plate. In fact, this type of implant was already developed by Biomet Sports Medicine (EZLoc; Warsaw, IN), and it is a very similar system; however, in the EZLoc, when the fixation plate is activated, it remains perpendicular to the tunnel and is locked, which does not allow repositioning in the longitudinal axis in cases involving opening of the plate inside the tunnel or in soft tissues. That system was used in our practice in 2005, and this technical difficulty (lack of control of the axis for cortical plate fixation) motivated the development of the ETD®, with wires connected in two holes, one at each end of the plate, making it freely moveable. The ETD® is a method for femoral fixation with a cortical suspension device, similar to the Endobutton. The advantages and disadvantages of each device are merely theoretical because there are no studies that compare them.

The fixation of the ETD® in soft tissues was observed in 6 of our 80 patients who underwent surgery. The first 5 of the 6 patients were treated with the transportal technique. After the 15th patient was treated with the transportal technique, we decided to routinely use radioscopy in all patients in the transportal group. Since then, we have had no more cases of ETD® fixation in soft tissues, and we had only one case in the transtibial group. In only 1 of the 6 patients, the ETD® migrated. Despite the migration, the patient did not develop knee instability. As reported, no significant difference was observed between patients with and without soft tissue fixation of the ETD®. Mae et al., in 2011, published a clinical study evaluating the fixation of the Endobutton in soft tissues [17]. In their sample, 25.2% of the cases exhibited these complication but no clinical impacts were reported. The authors used a double bundle graft, and they observed more interposition of the soft tissues in the posterolateral bundles. They suggested that this effect may have been due to the lateral femoral condyle anatomy, namely, the presence of ligament insertions and the close proximity of the iliotibial tract [17]. Our study also revealed more interpositions of the ETD® fixed distally and laterally in the femur, which occurred in the transportal cases, corroborating the hypothesis of the lateral femoral condyle anatomy as the cause of the problem.

We had only one case of ETD® fixation inside the femoral tunnel, and it occurred in the transtibial group. This complication is rare because when the wire is pulled to open the ETD® fixation plate and the device is inside the tunnel, the plate generally fails to open, indicating that the ETD® has not yet passed the lateral femoral cortex. Nevertheless, the patient exhibited good clinical evolution.

Rupture of the posterior cortex of the femoral tunnel was not observed in any case. In the transtibial group, using the guide in extension (Howell™ 65° Tibial Guide), which pre-sets the tibial tunnel angle and, thus, the positioning of the transtibial guide, prevented the occurrence of this complication. In the transportal group, we drilled the femoral tunnel with the knee in flexion (angle greater than 120°), avoiding the disruption of the posterior cortex. The higher the knee flexion, the greater the thickness of the wall of the femoral tunnel [18-20].

We considered a femoral tunnel to be short when it was less than 3.5 cm. We also considered the graft length inside the tunnel to be short when grafts were less than 2 cm. Both tunnel length and graft length inside the tunnel were significantly greater in the transtibial group. Despite this difference, the objective IKDC scores were not significantly different between patients with a short tunnel and those with a normal tunnel or between patients with small grafts in the tunnel and those with more than 2 cm of graft inside the tunnel. To prevent the occurrence of short tunnels when using the transportal technique, it is recommended that the tunnel be drilled with the knee in flexion at an angle greater than 120° [18-20].

A clinical evaluation comparing the groups is the aim of another study, in which the patients will be followed up for at least 2 years after surgery. The preliminary findings indicate that there was no significant difference in the KT1000, Lachman, pivot-shift, and anterior drawer test results between the two groups. Although more intercurrences were observed in the transportal group, the objective IKDC scores were significantly better in the transportal group compared with the transtibial group.

## Conclusions

The ETD® presented in this preliminary study has been shown to be a safe femoral fixation system that is easy to use and is associated with low numbers of peri- and postoperative intercurrences when used in conjunction with either the transtibial or transportal technique.

No author in this study has any conflicts of interest with the manufacturer or the seller of the ETD®; although one of the study authors collaborated in the device design, he and the other authors have never received any financial compensation or benefits from the companies involved.

## Consent

Written informed consent was obtained from all patients for the publication of this report and any accompanying images.

## References

[1] Emond CE, Woelber EB, Kurd SK, Ciccotti MG, Cohen SB (2011). A comparison of the results of anterior cruciate ligament reconstruction using bioabsorbable versus metal interference screws: a meta-analysis. J Bone Joint Surg Am.

[2] Milano G, Mulas PD, Ziranu F, Piras S, Manunta A, Fabbriciani C (2006). Comparison between different femoral fixation devices for ACL reconstruction with doubled hamstring tendon graft: a biomechanical analysis. Arthroscopy.

[3] Shen HC, Chang JH, Lee CH, Shen PH, Yeh TT, Wu CC, Kuo CL (2010). Biomechanical comparison of Cross-pin and Endobutton-CL femoral fixation of a flexor tendon graft for anterior cruciate ligament reconstruction–a porcine femur-graft-tibia complex study. J Surg Res.

[4] Oh YH, Namkoong S, Strauss EJ, Ishak C, Hecker AT, Jazrawi LM, Rosen J (2006). Hybrid femoral fixation of soft-tissue grafts in anterior cruciate ligament reconstruction using the EndoButton CL and bioabsorbable interference screws: a biomechanical study. Arthroscopy.

[5] Han DL, Nyland J, Kendzior M, Nawab A, Caborn DN (2012). Intratunnel versus extratunnel fixation of hamstring autograft for anterior cruciate ligament reconstruction. Arthroscopy.

[6] Stengel D, Casper D, Bauwens K, Ekkernkamp A, Wich M (2009). Bioabsorbable pins and interference screws for fixation of hamstring tendon grafts in anterior cruciate ligament reconstruction surgery: a randomized controlled trial. Am J Sports Med.

[7] Ma CB, Francis K, Towers J, Irrgang J, Fu FH, Harner CH (2004). Hamstring anterior cruciate ligament reconstruction: a comparison of bioabsorbable interference screw and endobutton-post fixation. Arthroscopy.

[8] Steiner M (2009). Anatomic single-bundle ACL reconstruction. Sports Med Arthrosc.

[9] Heming JF, Rand J, Steiner ME (2007). Anatomic limitations of transtibial drilling in anterior cruciate ligament reconstruction. Am J Sports Med.

[10] Steiner ME, Battaglia TC, Heming JF, Rang JD, Festa A, Baria M (2009). Independent drilling outperforms conventional transtibial drilling in anterior cruciate ligament reconstruction. Am J Sports Med.

[11] Sim JA, Gadikota HR, Li JS, Li G, Gill TJ (2011). Biomechanical evaluation of knee joint laxities and graft forces after anterior cruciate ligament reconstruction by anteromedial portal, outside-in, and transtibial techniques. Am J Sports Med.

[12] Bedi A, Musahl V, Steuber V, Kendoff D, Choi D, Allen AA, Pearle AD, Altchek DW (2011). Transtibial versus anteromedial portal reaming in anterior cruciate ligament reconstruction: an anatomic and biomechanical evaluation of surgical technique. Arthroscopy.

[13] Alentorn-Geli E, Samitier G, Alvarez P, Steinbacher G, Cugat R (2010). Anteromedial portal versus transtibial drilling techniques in ACL reconstruction: a blinded cross-sectional study at two- to five-year follow-up. Int Orthop.

[14] Hussein M, van Eck CF, Cretnik A, Dinevski D, Fu FH (2012). Prospective randomized clinical evaluation of conventional single-bundle, anatomic single-bundle, and anatomic double-bundle anterior cruciate ligament reconstruction: 281 cases with 3- to 5-year follow-up. Am J Sports Med.

[15] Anderson AF, Irrgang JJ, Kocher MS, Mann BJ, Harrast JJ (2006). The International Knee Documentation Committee Subjective Knee Evaluation Form: normative data. Am J Sports Med.

[16] Lysholm J, Gillquist J (1982). Evaluation of knee ligament surgery results with special emphasis on use of a scoring scale. Am J Sports Med.

[17] Mae T, Kuroda S, Matsumoto N, Yoneda M, Nakata K, Yoshikawa H, Shino K (2011). Migration of EndoButton after anatomic double-bundle anterior cruciate ligament reconstruction. Arthroscopy.

[18] Lubowitz JH (2009). Anteromedial portal technique for the anterior cruciate ligament femoral socket: pitfalls and solutions. Arthroscopy.

[19] Nakamura M, Deie M, Shibuya H, Nakamae A, Adachi N, Aoyama H, Ochi M (2009). Potential risks of femoral tunnel drilling through the far anteromedial portal: a cadaveric study. Arthroscopy.

[20] Gavriilidis I, Motsis EK, Pakos EE, Georgoulis AD, Mitsionis G, Xenakis TA (2008). Transtibial versus anteromedial portal of the femoral tunnel in ACL reconstruction: a cadaveric study. Knee.

